# Effect of Initial Surface Scratches on the Cavitation Erosion Behavior of 316L Stainless Steel Substrates and 316L Stainless Steel Coatings

**DOI:** 10.3390/ma16041392

**Published:** 2023-02-07

**Authors:** Pengfei Lu, Ziqi Xu, Ye Tian, Rui Yang, Kaixin Hu, Hua Li, Yanhong Yin, Xiuyong Chen

**Affiliations:** 1 Key Laboratory of Impact and Safety Engineering (Ministry of Education), School of Mechanical Engineering and Mechanics, Ningbo University, Ningbo 315211, China; 2 Zhejiang-Japan Joint Laboratory for Antibacterial and Antifouling Technology, Zhejiang Engineering Research Center for Biomedical Materials, Cixi Institute of Biomedical Engineering, Ningbo Institute of Materials Technology and Engineering, Chinese Academy of Sciences, Ningbo 315201, China; 3 Faculty of Materials Metallurgy and Chemistry, Jiangxi University of Science and Technology, Ganzhou 341000, China; 4 Chongyi Zhangyuan Tungsten Co., Ltd., Ganzhou 341000, China

**Keywords:** stainless steel coating, cavitation erosion, initial surface scratch, damage mechanism, plastic deformation, SEM observation

## Abstract

Rough surfaces have been widely considered as negative factors affecting cavitation erosion resistance. However, this study presented the opposite result. Here, 316L stainless steel substrates and the arc-sprayed 316L stainless steel coatings were subjected to a specific grinding process that introduced scratches on the surfaces. The surface hardness values of these ground specimens were measured to evaluate the influence of the grinding-induced strain hardening. The cavitation erosion performance of the specimens was evaluated. The results showed that rough surfaces with scratches could enhance the cavitation erosion resistance, particularly at the early stage of cavitation erosion. The scratches had a greater effect on the cavitation erosion resistance of the coatings than on the substrates. Moreover, rough surfaces with initial surface scratches could extend the incubation period of the 316L stainless steel substrates due to the inhibition of the plastic deformation. The SEM observation showed that the scratch structure of the coating surface inhibited the growth of cracks and the propagation of cavitation pits. This study could also serve as a reference for investigating the cavitation erosion behaviors of materials with a particular surface feature.

## 1. Introduction

Cavitation erosion is a typical surface failure involving complex physical and chemical processes [1]. It can cause severe wear in hydraulic machinery, such as pipelines, valves, and ship propellers [2,3]. Surface treatment techniques, such as laser surface treatment [4] and thermal spraying [5], have been widely employed to prepare anti-cavitation erosion coatings. Among the various techniques, thermal spraying methods have been extensively used to deposit coatings [6]. Until now, many kinds of coating materials, including engineering alloys [7], ceramics [8], and plastics [9], have been applied on metal alloy components to protect against cavitation erosion. Although many studies have been done on the cavitation erosion resistance of thermal sprayed coatings, the cavitation erosion mechanism of the coatings remains unclear.

It is well known that pores, cracks, oxides, and residual stress in thermal spray coatings have a great influence on cavitation erosion behavior [10,11]. For example, Wei et al. studied the effect of porosity on the cavitation erosion behavior by adding Cr_3_C_2_-NiCr to reduce the porosity of Ni-based composite coatings [12]. The results showed that pores are the preferential sites of cavitation erosion damage. Tian et al. sealed WC-10Co4Cr coatings using a polymer epoxy resin and investigated the effect of the sealant on the cavitation erosion behavior in a corrosive environment [5]. The results showed that the sealant penetration in the pores and cracks of the coatings not only effectively prevented crack growth during the cavitation erosion process but also significantly reduced the corrosion rate of the coatings. Silveira et al. prepared FeCrMnSiNi and FeCrMnSiB coatings with different oxide contents using high-velocity oxygen–fuel (HVOF) and high-velocity air–fuel (HVAF) methods [13]. The results showed that the higher cavitation erosion resistance could be related to the enhanced cohesive properties of splats in the thermal sprayed coatings with lower oxide contents. Varis et al. reported the effect of residual stress in the HVOF and HVAF sprayed WC-CoCr coatings on their cavitation erosion resistance [14]. The results showed that as a result of the high compressive residual stress in the coating, fatigue cracks were more difficult to form and were hindered in their development along lamellae interfaces, thereby improving the cavitation erosion resistance.

Additionally, the initial surface conditions can also affect the cavitation erosion resistance. It was reported that a rough surface with a high initial surface roughness could negatively affect the cavitation erosion resistance, resulting in extra material removal [15,16]. However, the cavitation erosion resistance of the arc-sprayed Fe-based amorphous/nanocrystalline coatings decreased with the increasing initial surface roughness [17]. Meanwhile, the literature also reported that the cavitation erosion resistance of the materials could be enhanced by introducing designed surface textures [18]. Gonzalez-Parra et al. showed that the laser-induced periodic surface structures (LIPSS) could mitigate cavitation erosion by reducing residual bubbles attached to their surfaces between cavitation events [19]. Nevertheless, very few studies have been reported on the influence of the initial surface features on the cavitation erosion behavior of the thermal sprayed coatings.

In this study, the influence of the initial surface scratches on the cavitation erosion behavior of thermal sprayed 316L stainless steel coatings was investigated. Both arc-sprayed 316L stainless steel coatings and 316L stainless steel substrates with scratches on the surface were prepared via a specific grinding procedure. The initial surface morphology of the specimens was measured using scanning electron microscopy (SEM) and a 3D optical profilometer. The phase composition and surface hardness values of the specimens before and after the grinding were investigated. The cavitation erosion properties of the specimens were evaluated in deionised water. Meanwhile, the evolution process of the specimens with initial surface scratches under cavitation erosion was studied via SEM observations. This study revealed that the initial surface features could play a dominant role in the material at the early stage of cavitation erosion. Meanwhile, the study also suggested that the configuration of the surface features could be a feasible method to enhance cavitation erosion resistance.

## 2. Experimental Section

### 2.1. Sample Preparation

The 316L stainless steel substrates with dimensions of Φ20 mm × 10 mm were used as the substrates. The arc spray (AS) system (EuTronic Arc Spray 4 HF, Castolin-Eutectic Pte Ltd., München, Germany) was employed to deposit the 316L stainless steel coatings on the substrates using Φ2 mm 316L stainless steel wires as the feedstock (Shijiazhuang Tong Bai Metal Technology Co., Ltd., Shijiazhuang, China). Prior to the spraying, the substrate surface was grit-blasted with 30-mesh alumina. Table 1 shows the arc spraying parameters.

The 316L stainless steel substrates and the 316L stainless steel coatings were ground with 80-, 400-, 800-, 1200-, and 2000-grit SiC paper and then polished with a 1 µm diamond suspension in an automatic grinding machine (SAPHIR 250 A1-ECO, QATM, Mammelzen, Germany). The polished 316L stainless steel substrates and the 316L stainless steel coatings were the control groups and were denoted as ‘316L’ and ‘coating’, respectively. Some polished specimens were re-ground by the automatic grinding machine using 800-grit SiC paper and the scratches were introduced. The re-ground 316L stainless steel substrates and the coatings were denoted as ‘ground 316L’ and ‘ground coating’, respectively. After the grinding process, the specimens were cleaned with deionised water and ethanol in an ultrasonic bath.

### 2.2. Sample Characterization

The surface morphologies of the specimens were studied via SEM (Regulus 8230, Hitachi, Japan). The porosity levels of the coatings were measured from three cross-sectional SEM images with a magnification of ×300 in ImageJ. The surface profiles were observed using a 3D optical profilometer (UP-Lambda, Rtec-Instruments Ltd., San Jose, CA, USA). The roughness was evaluated from the arithmetic mean height (Sa) of three different regions (1.9 mm × 1.2 mm) on the surface of each specimen. The phase compositions of the specimens before and after the grinding were studied by X-ray diffraction (XRD, D8 Advance, Bruker, Heidelberg, Germany) using Cu-Kα generated at 40 kV and 40 mA. The microhardness values of the specimens were measured using a Vickers hardness test (Wilson VH3300, Buehler, Reichshof, Germany) under a load of 0.2 kgf, and 25 measurements were taken at random sites for each specimen.

### 2.3. Cavitation Erosion Test

The cavitation erosion test was conducted using an ultrasonic vibratory device (GBS-SCT 20A, Guobiao Ultrasonic Equipment Co., Ltd., Hangzhou, China) according to ASTM G32-16 [20]. A schematic diagram of the cavitation erosion test system was shown in our previous study [21]. The test device consisted of a vibratory apparatus, a cooling system, and a control system. The rated power of the whole device was 1500 W. The vibration frequency and the peak-to-peak amplitude were 20 kHz and 50 ± 2.5 μm, respectively. The test medium was deionised water. The vibratory horn was immersed in the water at a depth of 23 ± 2 mm, and the distance of the specimen to the vibratory horn was 1 mm. The temperature was maintained at 25 ± 2 °C through a circulating cooling bath. The specimen was cleaned and dried in hot air after each test interval of 0.5 h. Then, the mass of the specimen was weighed using an electronic analytical balance (METTLER 220, TOLEDO Instruments Co., Ltd., Shanghai, China). Each specimen was weighed five times after each test interval, and three repeated tests were performed for each group of specimens. The specimens were tested for 6 h to ensure that the erosion rate was stabilized. The cumulative mass loss ($M_{loss}$) and the average erosion rate (${\dot{M}}_{loss}$) were used to estimate the cavitation resistance in this work. The $M_{loss}$ and ${\dot{M}}_{loss}$ were calculated according to the following equations, where M_0_ is the original mass, M_t_ is the mass after cavitation erosion, ΔM is the mass loss in the respective time interval, and Δt is the respective time interval:

(1) $$M_{loss}=M_{0}-M_{t}$$

(2) $${\dot{M}}_{loss}=\Delta M/\Delta t$$

Since the time interval for each group is 0.5 h, the equation can also be expressed as follows:

(3) $${\dot{M}}_{loss}=2*\Delta M$$

The damage evolution of the specimen surface during cavitation erosion was recorded by taking a series of SEM images at the same region at different intervals of the cavitation erosion test. All images were taken at the same magnifications, demonstrating the influence of the initial surface feature on the cavitation erosion behavior of the specimens.

## 3. Results and Discussion

**Figure 1:**
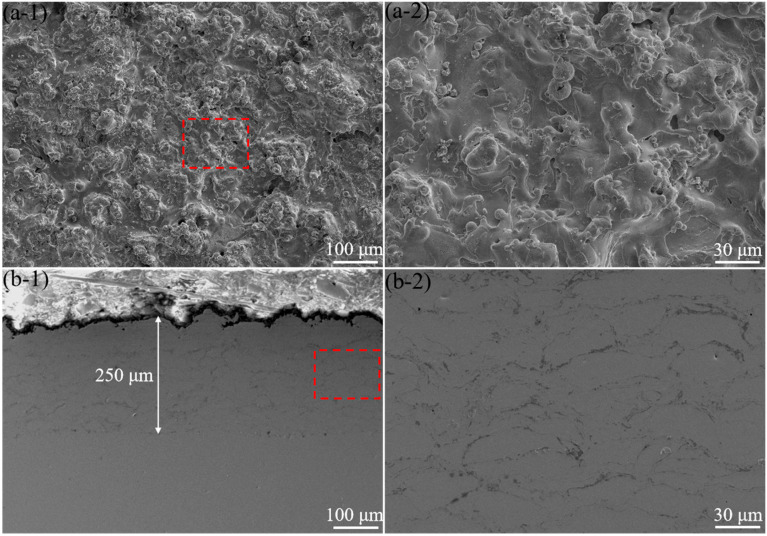
Surface (**a-1**,**a-2**) and cross-sectional (**b-1**,**b-2**) microstructures of the as-sprayed 316L stainless steel coating.

**Figure 2:**
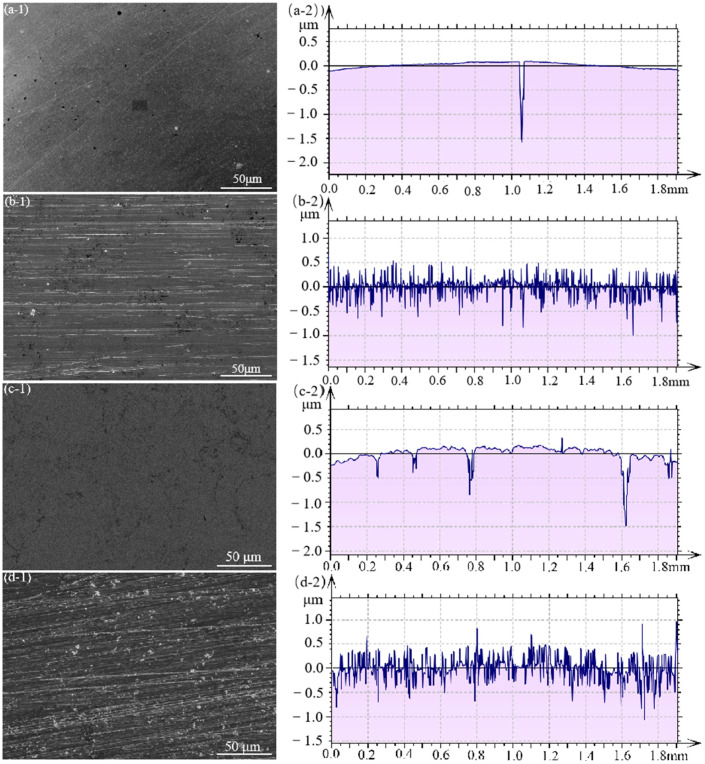
Surface (**-1**) and linear profiles (**-2**) of the 316L (**a**), ground 316L (**b**), coating (**c**), and ground coating (**d**) samples.

The SEM images of the surface and the cross-section of the as-sprayed 316L stainless steel coatings in Figure 1 showed the typical surface morphology of the sprayed coating (Figure 1a). The coating with a thickness of about 250 μm was successfully prepared, showing a lamellar microstructure (Figure 1b-1). Micron-sized pores were observed on the coating surface. Partially melted particles were also found (Figure 1b-2). In addition, the inter-splat boundaries could be clearly identified (Figure 1b-2), at which the cohesion strength may be weak. The average porosity of the 316L stainless steel coating was about 2.88%, which was consistent with previous reports [7,22].

The surface morphologies of the 316L, ground 316L, coating, and ground coating specimens before cavitation erosion are presented in Figure 2. For the polished samples, the surfaces of the 316L and coating specimens were relatively smooth (Figure 2a,c). After the grinding process, scratches were formed in the ground 316L and ground coating (Figure 2b,d). The surface of the ground 316L and ground coating became rough after the grinding process. The order of the samples ranging from rough to smooth started with the ground coating sample (Sa = 257 ± 85 nm), followed by the ground 316L (Sa = 145 ± 17 nm), coating (Sa = 122 ± 27 nm), and 316L (Sa = 65 ± 10 nm) samples.

**Figure 3:**
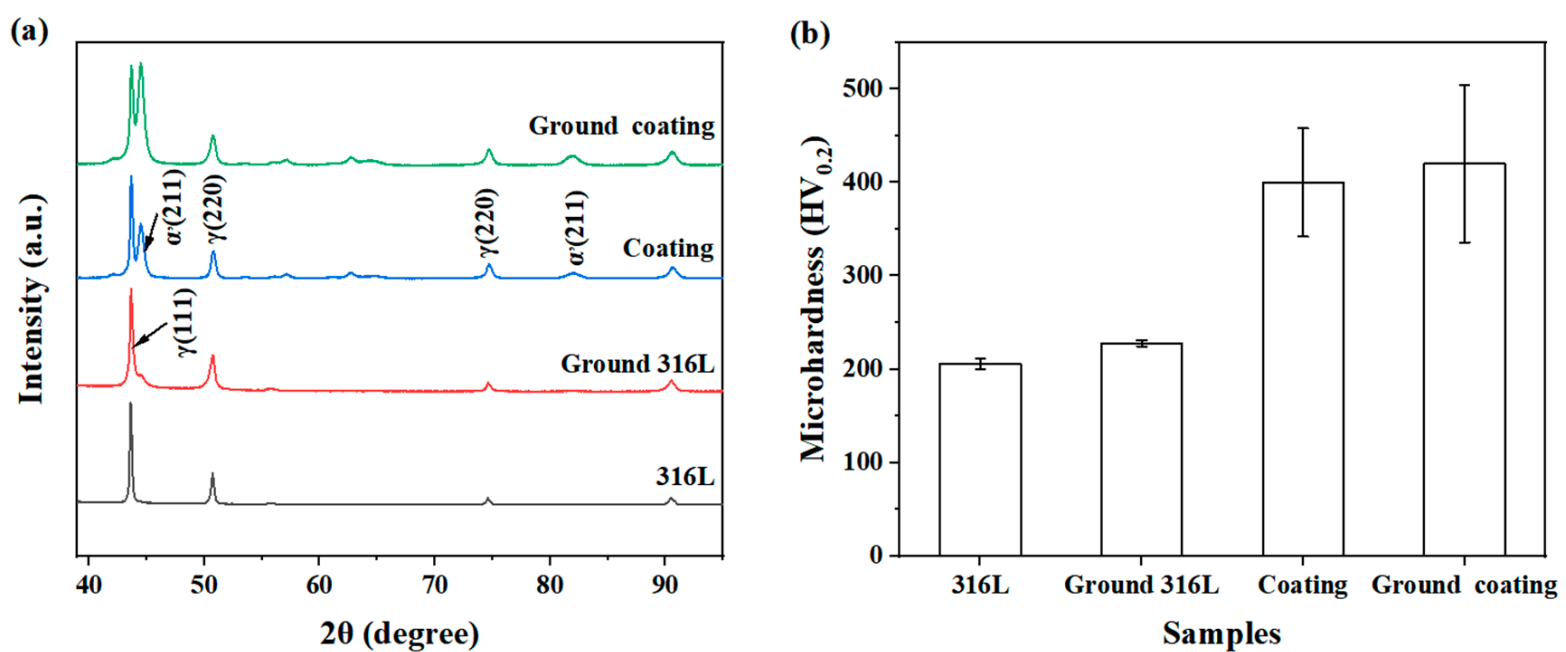
XRD patterns (**a**) and microhardness (**b**) values of the specimens before cavitation erosion.

According to the XRD patterns (Figure 3a), there was no significant difference in the chemical composition of the specimens before and after the grinding. It was reported that the cavitation erosion resistance could be enhanced with improved microhardness [23,24]. Therefore, the microhardness of the specimens was measured to evaluate the influence of the grinding process in this study. It can be seen that the average microhardness of the specimens was slightly increased by using a coarser grinding medium (Figure 3b), and the increase in microhardness was likely attributed to the grinding-induced hardening [25].

**Figure 4:**
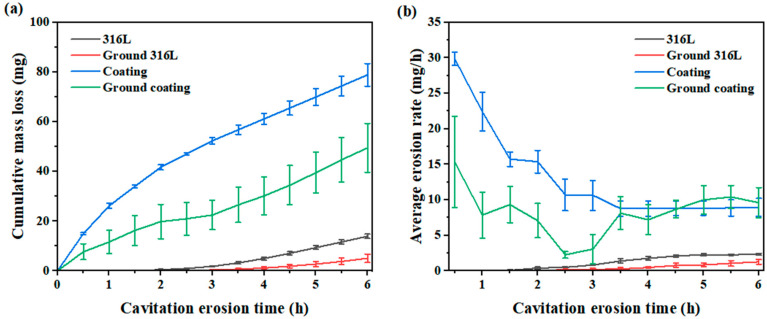
Cumulative mass losses (**a**) and average erosion rates (**b**) of the specimens after cavitation erosion exposure for 6 h in deionized water.

Figure 4 shows the cumulative mass losses and the average erosion rates of the specimens during cavitation erosion in deionised water at different test intervals. The cumulative mass losses of the ground 316L (5.1 ± 1.6 mg) and ground coating (49.5 ± 9.8 mg) specimens were significantly lower than those of the 316L (13.9 ± 0.9 mg) and coating (78.8 ± 4.6 mg) specimens (Figure 4a). As for the average erosion rates, similar results were observed during the first 3 h of erosion (Figure 4b). Gradually, the average erosion rates of the specimens stabilized with the increasing cavitation erosion time. After 6 h of cavitation erosion, the average order of the erosion rates of the four types of specimens was as follows: ground 316L < 316L < ground coating < coating. It is worth noting that the ground specimens with scratches exhibited better cavitation erosion resistance than the original polished specimens. Furthermore, the influence of scratches on the cavitation erosion resistance was higher for the coatings compared to the substrates. This phenomenon was because the pre-existing defects in the coating, such as the pores, cracks, and inter-splat boundaries with poor binding strength (Figure 1), were susceptible to cavitation attack. However, the bulk substrate was almost free of these defects. Therefore, the coated specimens were less resistant to cavitation erosion. For the coating specimens, the difference in the erosion rate was mainly observed at the initial stage of the cavitation erosion process (Figure 4b). Generally, materials with higher hardness are more resistant to cavitation erosion [26,27,28]. Despite the higher average microhardness of the ground coating than that of the coating sample, no significant difference was observed between them (Figure 3b). These results indicated that the grinding-induced strain hardening was not the dominant factor for the enhanced cavitation erosion resistance, since the initial surface features of these specimens were different. Thus, SEM observations were further employed to investigate the effects of the initial surface features on the damage mechanisms of these specimens during the cavitation erosion process.

**Figure 5:**
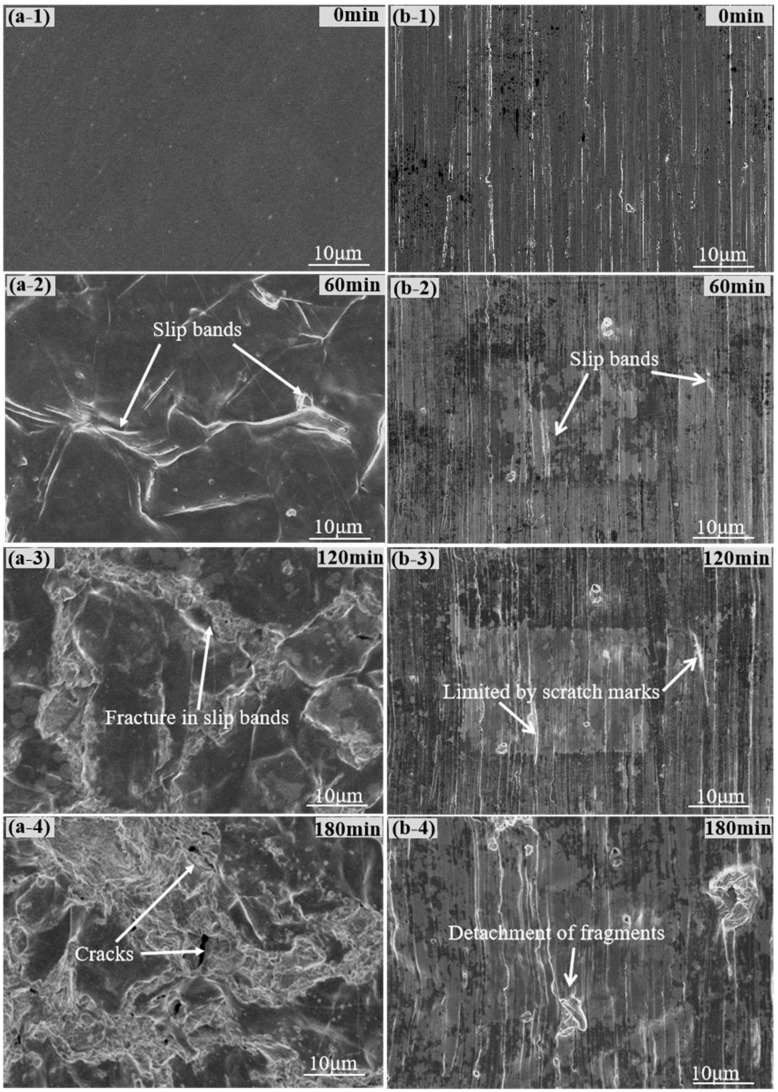
Surfaces of the 316L (**a-1**–**a-4**) and ground 316L (**b-1**–**b-4**) specimens before and after cavitation erosion.

The eroded surface morphologies of the 316L and ground 316L specimens after different erosion times are presented in Figure 5. After 60 min of cavitation erosion, slip bands were observed on the surface (Figure 5a-2), which were similar to the plastic deformation in the reported literature [29]. After 120 min of cavitation erosion, fractures in the slip bands were observed (Figure 5a-3). Therefore, it was suggested that the slip bands were preferentially damaged [30]. With the increase in erosion time, the original surface was gradually removed and cracks grew in the surface (Figure 5a-4). Based on the SEM observations (Figure 5a), the cavitation erosion proceeded in steps, including the accumulation of plastic deformation, the fractures in the slip bands, and the initiation and growth of cracks. These steps were associated with the fatigue fracture during cavitation erosion [31].

The effects of the initial surface feature (scratches) on the cavitation erosion behavior of the ground 316L sample are shown in Figure 5b. It is worth noting that compared to the 316L specimen (Figure 5a), the growth of slip bands in the ground 316L specimen was much slower, which contributed to the extended incubation period of the ground 316L specimen (Figure 4b). After 120 min of erosion, craters at the scratches were observed (Figure 5b-3) but were less severe than those in the 316L specimens. These craters slowly expanded when the ground 316L specimen was further exposed to cavitation erosion. After 180 min of erosion, the growth of the slip bands could still be observed on the eroded surfaces (Figure 5b-4). Since the incubation period represents the accumulation of plastic deformation, the growth of the slip bands suggested that the incubation period of the ground 316L specimens could be longer than 180 min. Through the SEM observation, the cavitation process of the ground 316L specimen was influenced by the scratches. The growth of the slip bands in the ground 316L specimen was hindered, resulting in an extended incubation period.

**Figure 6:**
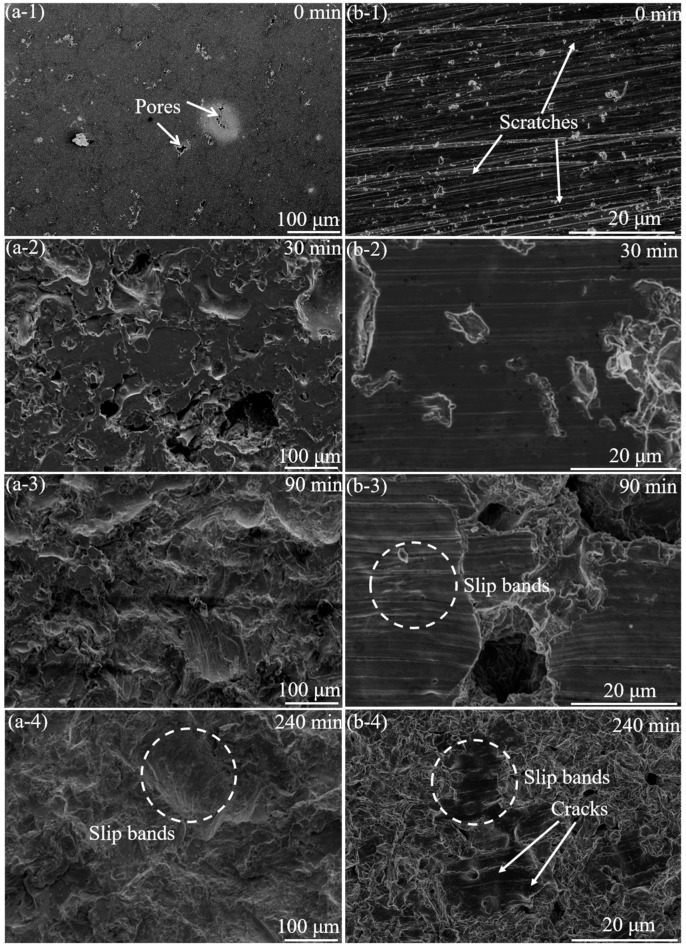
Surface of the coating (**a**) and ground coating (**b**) specimens before and after cavitation erosion.

SEM observations were also performed to obtain details of the cavitation erosion damage of the coating and ground coating specimens before and after 240 min of cavitation erosion testing in deionised water (Figure 6). Before the cavitation erosion test, pits were seen on the surface of the coating specimen (Figure 6a-1). Due to the porosity and weaker bonds at the inter-splat boundaries, the surface of the coating specimen was easily eroded. After 30 min of cavitation erosion, cavitation erosion craters were clearly visible on the eroded surface of the coating specimen (Figure 6a-2). When the cavitation erosion was performed for 90 min (Figure 6a-3), the original surface of the coating specimen was almost damaged, and a large area of the new surface was exposed. Plastic deformation (slip band) was observed on the newly exposed surface of the coating specimen. With the further prolongation of the cavitation erosion time, the original surface completely eroded and the plastic deformation further accumulated (Figure 6a-4).

For the ground coating specimen, after 30 min of cavitation erosion, relatively small cavitation pits and craters were observed on the surface of the ground coating specimen and were likely induced by original defects such as pores (Figure 6b-2). When the cavitation erosion was carried out for 90 min, large cavitation craters and plastic deformation were observed (Figure 6b-3). Compared to the coating specimen, the original surface of the ground coating specimen was relatively intact. Stress accumulation produced deformation, and when the surface stress accumulated to a certain extent, fatigue fracture occurred on the surface and cracks were present [22]. When the cavitation erosion was carried out for 240 min (Figure 6b-4), due to the continuous accumulation of stress, the expansion of the cavitation pit on the ground coating specimen caused a great deal of peeling of the original surface. Nevertheless, it is worth noting that the original surface of the ground coating specimen was not completely eroded, which differed from that of the coating specimen.

**Figure 7:**
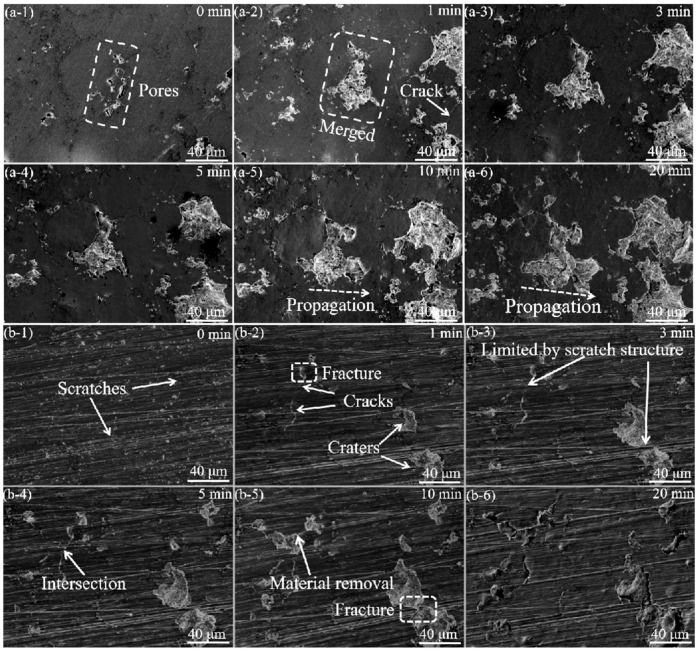
The microstructural evolution of the coating (**a**) and ground coating (**b**) specimens during the cavitation erosion process for (**-1**) 0 min, (**-2**) 1 min, (**-3**) 3 min, (**-4**) 5 min, (**-5**) 10 min, and (**-6**) 20 min.

The SEM observation was further performed on the coating and ground coating specimens to investigate the morphological changes on the surfaces at the early stages of cavitation erosion. As shown in Figure 7, the failure process of the ground coating was different from that of the coating. The pores acted as vulnerable sites in the coating, which would be preferentially damaged during the surface erosion process (Figure 7a). Craters were formed during the first 1 min of the erosion (Figure 7a-2). With the increase in cavitation erosion time, the existing craters tended to propagate and merge with the adjacent craters (Figure 7a). Although small craters were also observed on the ground coating after 1 min of cavitation erosion, the propagation of the erosion was significantly inhibited by the scratches (Figure 7b). After 10 min of cavitation erosion, the craters grew larger (Figure 7b-5). Then, these craters expanded towards the adjacent scratches. The SEM observation indicated that initial surface scratches could hinder the propagation of erosion and change the failure mode of the ground coating exposed to cavitation erosion.

**Figure 8:**
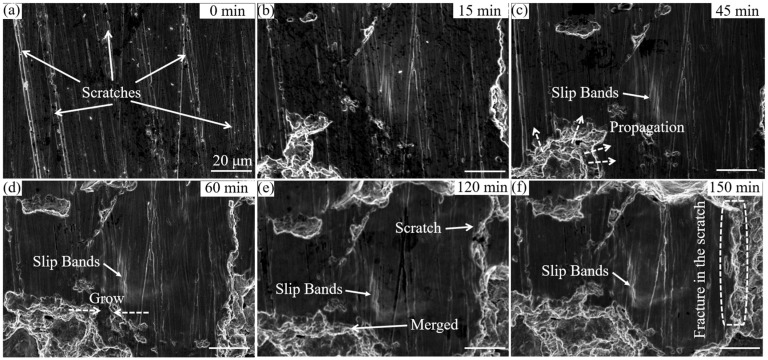
The microstructural evolution of the ground coating during the cavitation erosion process for (**a**) 0 min, (**b**) 15 min, (**c**) 45 min, (**d**) 60 min, (**e**) 120 min, and (**f**) 150 min.

To further investigate the effect of the initial scratches on the cavitation erosion behavior of the ground coating sample, a longer SEM observation experiment was performed (Figure 8). After 15 min of cavitation erosion, slip bands appeared between the surface scratches and cavitation craters were also observed on the surface of the sample (Figure 8b). After 45 min of cavitation erosion, the stress accumulated further at the slip zone and the cavitation craters continued to expand to the surroundings (Figure 8c). As the cavitation erosion time was prolonged, the cavitation craters further expanded and merged to form a larger cavitation crater (Figure 8d,e). It was also observed that the expansion of cavitation craters to the scratches was inhibited and the scratches were detached, which greatly reduced the mass loss rate of the material surface. Compared with the coating, besides the slight increase in microhardness, the restriction of the scratches on the propagation of erosion was the possible reason for the relatively lower mass loss after 240 min of erosion in the ground coating.

Generally, the surface roughness can alter the flow dynamics and change the cavitation erosion behaviors. For example, the entrapped gas and liquid in cavitation craters can damp the incoming cavitation pressure waves [32]. It was also reported that rough surfaces could reduce the bubble size during acoustic cavitation [33]. Hence, it can be assumed that specimens with scratches may achieve a similar damping effect via entrapping gas or liquid in these scratches and reducing the bubble size during acoustic cavitation. Therefore, the differences in the cavitation erosion performances of these specimens may be explained based on this assumption. Moreover, the samples underwent work hardening during the grinding process, resulting in a slight increase in Vickers hardness, which was beneficial in preventing cavitation damage [34]. According to previous reports, work hardening occurs on the bottom and edges of scratches [35], resulting in martensitic transformation (Figure 3a) and grain refinement [36,37,38]. In samples with initial surface scratches, the higher hardness in the areas with scratches inhibited crack propagation when the cracks expanded to the scratches (Figure 7b and Figure 8). Thus, the samples with initial surface scratches showed much reduced mass losses during cavitation erosion due to their inhibitory effect on the crack propagation (Figure 4). Nevertheless, further efforts are needed to investigate the effects of the initial surface scratches on the cavitation erosion behavior of the materials.

## 4. Conclusions

In this study, the influence of the initial surface scratches on the cavitation erosion resistance of 316L stainless steel substrates and 316L stainless steel coatings was investigated. The damage mechanism of the 316L stainless steel substrates and 316L stainless steel coatings with initial surface features in response to cavitation erosion were explored using SEM observations. Based on the results, the following conclusions can be drawn:

(1) Both the ground 316L and ground coating specimens with initial surface scratches performed better in terms of cavitation erosion resistance at the early stage of the cavitation erosion compared to the 316L and coating specimens without initial surface scratches;

(2) In addition, the effect of the scratches on the cavitation erosion resistance was higher for the coatings compared to the substrates. This was possibly due to the fact that the pre-existing defects in the coating were vulnerable to cavitation attack, while the bulk substrate was almost defect-free;

(3) The initial surface scratches could hinder the propagation of erosion and change the failure mode of ground substrates and ground coatings exposed to cavitation erosion.

## Figures and Tables

**Table 1:** Arc spraying parameters.

Parameters	Values
Voltage (V)	33
Current (A)	280
Wire feed rate (m/min)	4.7
Compressed air pressure (bar)	6
Spraying distance (mm)	200

## Data Availability

All data are available in the main text.

## Abbreviations

AS	Arc spray
HVAF	High-velocity air–fuel
HVOF	High-velocity oxygen–fuel
LIPSS	Laser-induced periodic surface structures
*M_loss_*	The cumulative mass loss
*S_a_*	The arithmetic mean height
SEM	Scanning electron microscopy
XRD	X-ray diffraction

## References

[1] Karimi A., Martin J. (1986). Cavitation erosion of materials. Int. Mater. Rev..

[2] Kamoleka M., Xu H., Sun F., Wang H., Madenge G. (2020). Material analysis and molecular dynamics simulation for cavitation erosion and corrosion suppression in water hydraulic valves. Materials.

[3] Yusvika M., Prabowo A.R., Baek S.J., Prija Tjahjana D.D.D. (2020). Achievements in observation and pre-diction of cavitation: Effect and damage on the ship propellers. Procedia Struct. Integr..

[4] Tian Y., Yang R., Gu Z., Zhao H., Wu X., Dehaghani S.T., Chen H., Liu X., Xiao T., McDonald A. (2022). Ultrahigh cavitation erosion resistant metal-matrix composites with biomimetic hierarchical structure. Compos. B Eng..

[5] Tian Y., Zhang H., Chen X., McDonald A., Wu S., Xiao T., Li H. (2020). Effect of cavitation on corrosion behavior of HVOF-sprayed WC-10Co4Cr coating with post-sealing in artificial seawater. Surf. Coat. Technol..

[6] Zhang H., Yongfeng G., Chen X., McDonald A., Li H. (2019). A comparative study of cavitation erosion resistance of several HVOF-sprayed coatings in deionized water and artificial seawater. J. Therm. Spray Technol..

[7] Wang Z., Zhang X., Cheng J., Lin J., Zhou Z. (2014). Cavitation erosion resistance of Fe-based amorphous/nanocrystal coatings prepared by high-velocity arc spraying. J. Therm. Spray Technol..

[8] Jonda E., Szala M., Sroka M., Łatka L., Walczak M. (2023). Investigations of cavitation erosion and wear re-sistance of cermet coatings manufactured by HVOF spraying. Appl. Surf. Sci..

[9] Li N., Yang R., Tian Y., Lu P., Huang N., Li H., Chen X. (2023). Synthesis of durable hydrophobic fluorinated polyurethanes with exceptional cavitation erosion resistance. Tribol. Int..

[10] Hauer M., Gärtner F., Krebs S., Thomas K., Makoto W., Seiji K., Werner K., Knuth-Michael H. (2021). Process selection for the fabrication of cavitation erosion-resistant bronze coatings by thermal and kinetic spraying in maritime applications. J. Therm. Spray Technol..

[11] Liu Y., Liu W., Ma Y., Meng S., Liu C., Long L., Tang S. (2016). A comparative study on wear and corrosion behaviour of HVOF- and HVAF-sprayed WC–10Co–4Cr coatings. Surf. Eng..

[12] Wei X., Ban A., Zhu W., Zhu D., Xu J., Zhang C. (2021). Cavitation erosion resistance of TiNi-based composite coating deposited by APS. J. Therm. Spray Technol..

[13] Silveira L., Pukasiewicz A.G.M., de Aguiar D.J.M., Zara A.J., Björklund S. (2019). Study of the corrosion and cavitation resistance of HVOF and HVAF FeCrMnSiNi and FeCrMnSiB coatings. Surf. Coat. Technol..

[14] Varis T., Suhonen T., Laakso J., Jokipii M., Vuoristo P. (2020). Evaluation of residual stresses and their influence on cavitation erosion resistance of high kinetic HVOF and HVAF sprayed WC-CoCr coatings. J. Therm. Spray Technol..

[15] Ahmed M.S., Hokkirigawa K., Kikuchi K., Higuchi J., Oba R. (1993). SEM studies of particles produced by cavitation erosion. JSME Int. J. Ser. B.

[16] Lin C., Chen K., He J. (2006). The cavitation erosion behavior of electroless Ni–P–SiC composite coating. Wear.

[17] Lin J., Wang Z., Cheng J., Kang M., Fu X., Hong S. (2017). Effect of initial surface roughness on cavitation erosion resistance of arc-sprayed Fe-based amorphous/nanocrystalline coatings. Coatings.

[18] Lin N., Li D., Zou J., Xie R., Wang Z., Tang B. (2018). Surface texture-based surface treatments on Ti6Al4V titanium alloys for tribological and biological applications: A mini review. Materials.

[19] Gonzalez-Parra J.C., Robles V., Devia-Cruz L.F., Rodriguez-Beltran R.I., Cuando-Espitia N., Camacho-Lopez S., Aguilar G. (2022). Mitigation of cavitation erosion using laser-induced periodic surface structures. Surf. Interfaces.

[20] (2016). Standard Test Method for Cavitation Erosion Using Vibratory Apparatus.

[21] Zhang H., Chen X., Gong Y., Tian Y., McDonald A., Li H. (2020). In-situ SEM observations of ultrasonic cavitation erosion behavior of HVOF-sprayed coatings. Ultrason. Sonochem..

[22] Lin J., Wang Z., Cheng J., Kang M., Fu X., Hong S. (2019). Evaluation of cavitation erosion resistance of arc-sprayed Fe-based amorphous/nanocrystalline coatings in NaCl solution. Results Phys..

[23] Chiu K., Cheng F., Man H.C. (2005). Cavitation erosion resistance of AISI 316L stainless steel laser surface-modified with NiTi. Mater. Sci. Eng. A.

[24] Pukasiewicz A., De Boer H., Sucharski G., Vaz R., Procopiak L. (2017). The influence of HVOF spraying parameters on the microstructure, residual stress and cavitation resistance of FeMnCrSi coatings. Surf. Coat. Technol..

[25] Poggie R., Wert J. (1991). The influence of surface finish and strain hardening on near-surface residual stress and the friction and wear behavior of A2, D2 and CPM-10V tool steels. Wear.

[26] Szala M., Łatka L., Awtoniuk M., Winnicki M., Michalak M. (2020). Neural modelling of aps thermal spray process parameters for optimizing the hardness, porosity and cavitation erosion resistance of Al_2_O_3_ -13 wt% TiO_2_ coatings. Processes.

[27] Szala M., Walczak M., Świetlicki A. (2021). Effect of microstructure and hardness on cavitation erosion and dry sliding wear of HVOF deposited CoNiCrAlY, NiCoCrAlY and NiCrMoNbTa coatings. Materials.

[28] Lin J., Wang Z., Lin P., Cheng J., Zhang X., Hong S. (2014). Microstructure and cavitation erosion behavior of FeNiCrBSiNbW coating prepared by twin wires arc spraying process. Surf. Coat. Technol..

[29] Gao G., Zhang Z. (2019). Cavitation erosion behavior of 316L stainless steel. Tribol. Lett..

[30] Bregliozzi G., Di Schino A., Ahmed S.-U., Kenny J., Haefke H. (2005). Cavitation wear behaviour of austenitic stainless steels with different grain sizes. Wear.

[31] Hattori S., Nakao E. (2001). Cavitation erosion mechanisms and quantitative evaluation based on erosion particles. Wear.

[32] Chahine G.L., Franc J.-P., Karimi A. (2014). Mass loss and advanced periods of erosion. Advanced Experimental and Numerical Techniques for Cavitation Erosion Prediction.

[33] Altay R., Sadaghiani A.K., Sevgen M.I., Şişman A., Koşar A. (2020). Numerical and experimental studies on the effect of surface roughness and ultrasonic frequency on bubble dynamics in acoustic cavitation. Energies.

[34] Selvam K., Mandal P., Grewal H.S., Arora H.S. (2018). Ultrasonic cavitation erosion-corrosion behavior of friction stir processed stainless steel. Ultrason. Sonochem..

[35] Xu X., van der Zwaag S., Xu W. (2015). A novel multi-pass dual-indenter scratch test to unravel abrasion damage formation in construction steels. Wear.

[36] Nakada N., Ito H., Matsuoka Y., Tsuchiyama T., Takaki S. (2010). Deformation-induced martensitic transformation behavior in cold-rolled and cold-drawn type 316 stainless steels. Acta Mater..

[37] Tian Y., Zhao H., Yang R., Liu X., Chen X., Qin J., McDonald A., Li H. (2022). In-situ SEM investigation on stress-induced microstructure evolution of austenitic stainless steels subjected to cavitation erosion and cavitation erosion-corrosion. Mater. Des..

[38] Pereira J., Tressia G., Machado P., Franco L., Sinatora A. (2018). Scratch test of pearlitic steels: Influence of normal load and number of passes on the sub-superficial layer formation. Tribol. Int..

